# Semantic annotation of morphological descriptions: an overall strategy

**DOI:** 10.1186/1471-2105-11-278

**Published:** 2010-05-25

**Authors:** Hong Cui

**Affiliations:** 1 School of Information Resources and Library Science, University of Arizona, 1515 E. First Street, Tucson Arizona, 85719 USA

## Abstract

**Background:**

Large volumes of morphological descriptions of whole organisms have been created as print or electronic text in a human-readable format. Converting the descriptions into computer- readable formats gives a new life to the valuable knowledge on biodiversity. Research in this area started 20 years ago, yet not sufficient progress has been made to produce an automated system that requires only minimal human intervention but works on descriptions of various plant and animal groups. This paper attempts to examine the hindering factors by identifying the mismatches between existing research and the characteristics of morphological descriptions.

**Results:**

This paper reviews the techniques that have been used for automated annotation, reports exploratory results on characteristics of morphological descriptions as a genre, and identifies challenges facing automated annotation systems. Based on these criteria, the paper proposes an overall strategy for converting descriptions of various taxon groups with the least human effort.

**Conclusions:**

A combined unsupervised and supervised machine learning strategy is needed to construct domain ontologies and lexicons and to ultimately achieve automated semantic annotation of morphological descriptions. Further, we suggest that each effort in creating a new description or annotating an individual description collection should be shared and contribute to the "biodiversity information commons" for the Semantic Web. This cannot be done without a sound strategy and a close partnership between and among information scientists and biologists.

## Background

**Figure 1:**
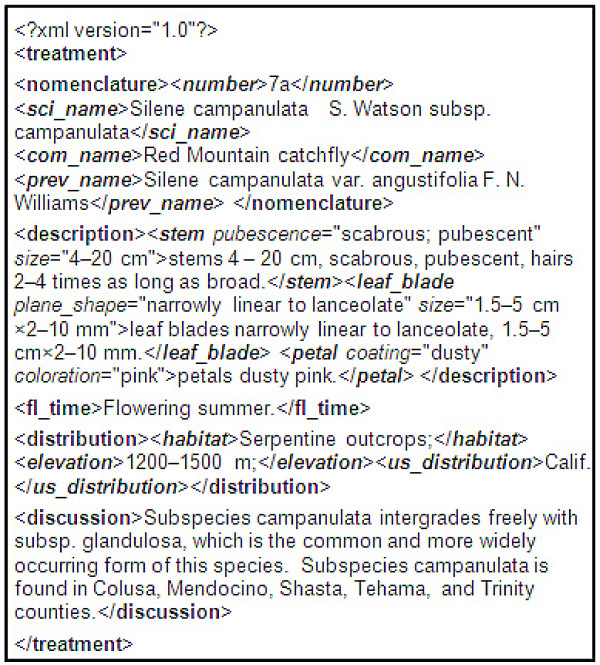
**An annotated morphological description**. "<>" enclosed text is a tag. Bold font represents paragraph level annotation, bold and italic clause level annotation, and italic character level annotation. Annotation produced by an annotation system created for FNA by the author.

Converting free text morphological descriptions of whole organisms into a computer-readable representation where organ names and characters are explicitly marked with meaningful tags promises more effective use of biodiversity knowledge and better support for biodiversity research. The conversion task is commonly called "semantic markup" or "semantic annotation." Here "semantic" means each concept is assigned one and only one unambiguously defined meaning. Due to the volume of the documents, automatic procedures are needed to perform the task. Semantic annotation research has been very active and conducted in various domains. The domain of morphological descriptions presents rather distinctive characteristics. Figure 1 shows an annotated plant description in XML (eXtensible Markup Language) format.

Semantic annotation of morphological descriptions may be at "clause" or "character" level. A clause is a segment of text terminated by a semicolon (;) or period (.). In morphological descriptions, a clause may not be a grammatical sentence (see examples in Figure 1). Clause-level annotation labels individual clauses with a meaningful tag, while character-level annotation identifies character/state pairs for describing organs. In addition, a method distinguishing description paragraphs for nomenclature, distribution, and other types of sections is also needed. Figure 1 illustrates the three levels of annotation in XML for a plant description from the Flora of North America [1].

The inserted tags bring a computer's "understanding" of morphological descriptions to a higher level that would support more intelligent usages of the information than keyword-based search. Besides improving the accuracy of information retrieval, the tags make it possible for a computer to quickly merge or compare different descriptions organ by organ and character by character. This new capability will impact comparative biological research, the methods used in generating identification keys, and even the way an editor reviews manuscripts [2,3].

Relevant research in annotating biosystematic literature will be reviewed next.

## Methods

### Methods used for semantic annotation of taxonomic documents

A syntactic parsing technique was used by a number of earlier projects. Taylor and Abascal & Sanchenz hand-crafted a set of simple grammar rules and a small lexicon specifically for extracting character states from several Floras [4,5]. Taylor's performances were not scientifically evaluated but estimated at 60% to 80% recall.

The major advantage of the syntactic parsing approach lies in the ease of constructing a parser once the lexicon and grammar rules are prepared. The main drawback is precisely its reliance on the lexicon and grammar rules. Because of the diverse terminologies and the deviated syntax from natural language (see Characteristics of morphological descriptions in the Results section), preparing lexicons and grammar rules for each individual collection or taxon group would be prohibitively expensive.

Rules called "regular expression patterns" that rely on the regularity in the style and the use of punctuation marks were hand-crafted and found to be useful for extracting nomenclature and distribution information [6-8]. However, this approach is not useful for morphological descriptions, because of (1) the lack of such regularity in morphological descriptions and (2) the low reusability of such rules on a different description collection. Lydon et al. showed that the narratives on five common species were so different among six English Floras that only 9% of information was expressed in the same way [9].

**Figure 2:**
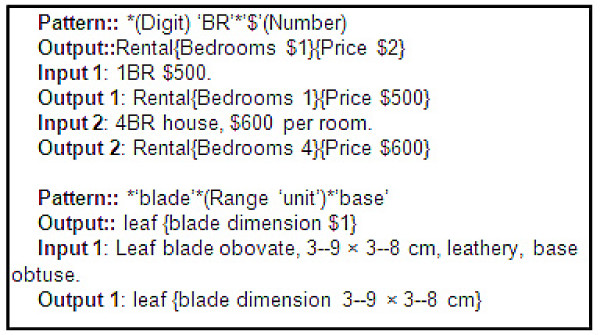
**Two regular expression patterns**. The first (Soderland, 1999) is for extracting bedroom number and rent from apartment rental ads. The pattern extracts the digit before "BR" as the number of bedrooms ($1) and the number after a "$" as the rent ($2). The pattern produces the correct result for Input 1 but a wrong result for Input 2, as $600 was the price for one room, not four rooms. The pattern will not match or extract anything from "1 large BR $500" or "1 master BR $500." The second (Tang & Heidorn, 2007) extracts leaf blade dimension by looking for a range between the words "blade" and "base."

Regular expression patterns may be generated automatically using the supervised machine learning technique, where an algorithm uses the occurrences of different patterns in training examples to statistically predict the pattern that is likely to fit a new example. Soderland developed such an algorithm to extract information from semi-structured documents such as apartment rental ads [10]. Figure 2 shows such an extraction pattern. This algorithm was adapted by Tang & Heidorn to extract leaf shape, size, color, arrangement, and fruit/nut shape from 1600 FNA species descriptions [2]. They reported the extraction accuracies ranging from 30% to 100%.

Supervised learning technique was also used by Cui, in which the algorithm performed clause level annotation by learning from training examples what are called "association rules," which are less sensitive to text variations, compared to the extraction pattern discussed above [11]. Its annotation accuracy ranged between upper 80% to upper 90% on three different Floras (Flora of North America, Flora of China, and Flora of North Central Texas) of over ten thousand descriptions [1,12-15].

Machine learning has the advantage over manual work in its ability to programmatically evaluate its learning and adjust candidate patterns/rules based on what is seen in the training examples. However, the need for training examples is also a shortcoming, as training examples must be prepared for different taxon groups and even different collections. More importantly, if certain organs/characters are not included in the predefined extraction targets, they will be quietly ignored, resulting in loss of information. This "inadequate template" problem was also noted by Wood et al., where manually-created dictionaries, an ontology, and a lookup list were used to extract and correlate characters/states from a set of 18 plant species descriptions [16]. They had to tag organs that were not in their lists "UnknownPlantPart." Wood et al. used parallel text to find three times more targeted information, which would otherwise be missed, and improved extraction recall three times. Diederich, Fortuner & Milton reported a system called Terminator, which is very similar to Wood et al.'s in that they both use a hand-crafted domain ontology to support character extraction [17].

The previous approaches share the inadequate template problem because a fixed template cannot be expected to cover the diverse terms in morphological descriptions well. Manually adding new terms to the templates has been suggested as a way to solve the problem [16]. Table 1 summarizes the techniques reviewed. The performances reported may not be comparable, as different evaluation schemes were used.

**Table 1:** Review of the existing annotation techniques.

Methods	Handmade prerequisites and their reusability	Annotation Level	Results and their reusability	Scope of evaluation	Performance (*)
**Syntactic parsing:** **1. Abascal & Sanchenz (1999)** **2. Taylor (1995)**	Lexicon & grammar rules: Not good for another taxon group/collection.	1. Paragraph 2. Character	1. Style clues: Less reusable. 2. Organ names & character states: Reusable.	1. FNA v. 19 2. Flora of New South Wales, Flora of Australia.	1. Not reported 2. Roughly estimated recall:60%-80%
**Supervised machine learning--text classification: Cui & al. (2002)**	Training examples: Not good for another taxon group.	paragraph	Classification models: Less reusable.	1500+ descriptions from FNA	Recall: 94% Precision: 97%
**Ontology based extraction:** **1. Diederich, Fortuner & Milton (1999)** **2. Wood & al. (2003)**	Dictionaries, ontology, & checklists: Not good for another taxon group.	Character	Organ names & character states: Reusable.	1. 16 descriptions 2. 18 species descriptions from six Floras.	1. Accuracy on 1 sample:76% 2. Recall: 66% Precision: 74%
**Supervised machine learning--extraction patterns: Tang & Heidorn (2007)**	Extraction template & training examples: Not good for another taxon group.	Character, limit to these character states: leaf shape, size, color; Fruit type.	Extraction patterns: Sensitive to text variations, less reusable. Character states: Reusable.	1600 FNA species descriptions.	Recall: 33%-80% Precision:75%-100%
**Supervised machine learning--** **association rules: Cui (2008a)**	Annotation template & training examples: Not good for another taxon group.	Clause	Association rules: Reusable only within the same taxon group	16,000 descriptions from FNA, FOC, and FNCT	Recall and precision: 80%-95%
**Unsupervised learning: Cui (2008b)**	No prerequisites	1. Clause 2. Character	Organ names & character states: Reusable.	FNA, FOC, & Treatises Part H	Precision 88-95% Recall 50%-75%

* Precision is the proportion of the computer's decisions that is correct. Recall is the proportion of all targets correctly discovered by the computer.

The techniques reviewed here all have their strengths, despite weaknesses, yet, when facing the millions of OCRed text descriptions produced by the Biodiversity Heritage Library, none of them seems to be both effective and efficient [18]. The reason, we suggest, lies in the special characteristics of the morphological descriptions.

## Results

In this section we present our exploratory results on the characteristics of morphological descriptions and on an unsupervised machine learning strategy. Insights gained via these exercises give rise to an overall strategy for semantic annotation of morphological descriptions, which we shall discuss at the end of this section.

### Characteristics of morphological descriptions

The performance of a semantic annotation technique depends, at least in part, on the characteristics of the documents to be annotated. A technique that identifies organ names by looking for bold words, for example, is not very useful for the task overall, because many descriptions are not styled that way. Here, in our search for a sound overall strategy to mark up all morphological descriptions in English, we consider some general characteristics of morphological descriptions which are challenging or beneficial for an automated semantic annotation technique.

#### 1. Challenging characteristics

Diverse terminology: each biodiversity branch has a more or less distinct set of terminology. Not only are terms used in brachiopod (Animalia) descriptions different from those in plant descriptions, but terms in one plant family description are somewhat different from those in another. Several previous researchers (e.g. Wood et al., and Cui & Heidorn) have reported that when applying a system crafted from one set of documents to a different set, new concepts that were unknown to the system were encountered, forcing an automated system to work in an interactive and iterative fashion to incorporate new concepts along the way [16,19].

**Figure 3:**
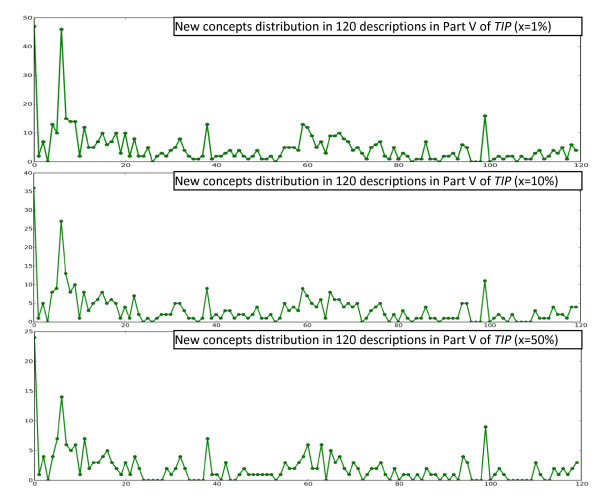
**The counts of new domain concepts in Part V of TIP using different sized common word filters**.

**Figure 4:**
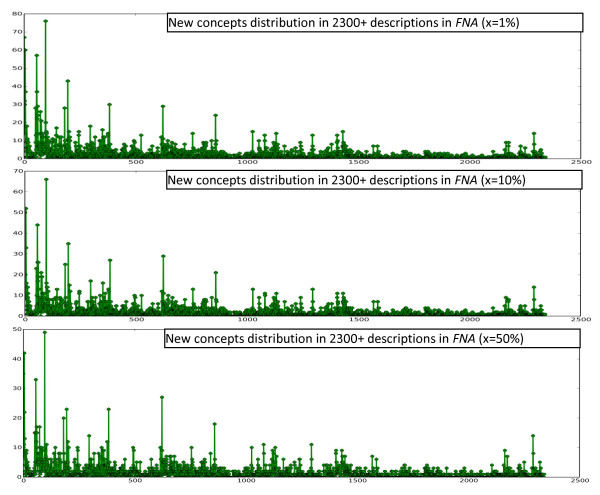
**The counts of new domain concepts in FNA using different sized common word filters**.

**Figure 5:**
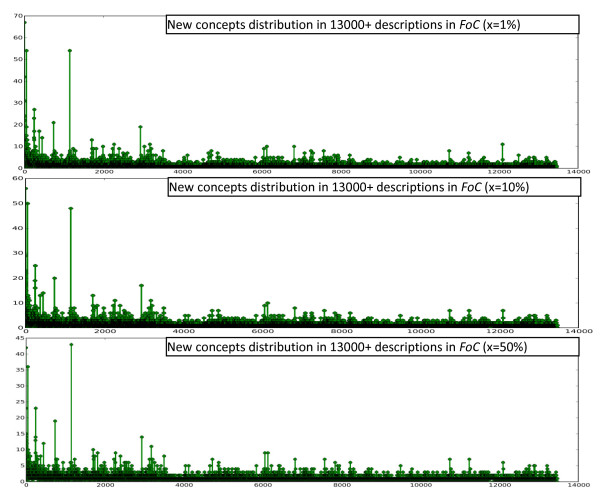
**The counts of new domain concepts in FOC using different sized common word filters**.

To find out how biodiversity concepts are distributed in description collections, a simple procedure was used on several collections of morphological descriptions of different taxa and different size, including 120 descriptions from Part V of Treatise on Invertebrate Paleontology (TIP), 2300 descriptions from Flora of North America (FNA), and 13,000 descriptions from Flora of China (FOC). Concepts collected from the collections are included in the Additional Files 1, 2, and 3. The procedure involves using the Brown Corpus to filter out non-domain concepts and then having the computer read the descriptions one by one in the order the descriptions are presented in the original publications [20]. In our experiments, the top x percent of the most frequent words were taken from all sections of Brown Corpus, except for section "J: Learned," to form a set of most common non-technical terms in English. Not counting section "J," the Brown Corpus contains 979,304 words, of which 42,262 are unique. Words appearing in morphological descriptions but not in this set were considered domain concepts, which may include organ names, characters, and character states (lists of extracted domain concepts, with x set to 10%, from the three sources are included in Additional Files). We used three settings for x: x = 1%, 10%, or 50%. The computer recorded the number of domain concepts in a description that were seen for the first time while reading the descriptions one by one. The resulting plots using different x and description sources are shown in Figures 3, 4, and 5. The plots suggest that new concepts are constantly encountered regardless of the size of a collection and the size of the common word filter. In other words, systems built based on a sample of a collection will encounter new concepts constantly when used on the remaining part of the collection. The diverse terminologies and the absence of a comprehensive computer-readable dictionary/lexicon covering all these terminologies present a challenge for automated semantic annotation systems, because a) words are the basic unit in language processing--a higher level of understanding of the text cannot be obtained without a good understanding of the words and b) this characteristic makes a system crafted for one (or portion of a) description collection easily fail on the new concepts contained in another (or another portion of the) collection.

Diverse meanings: While it is well-known that the same word could have different meanings in different domains, the exact meaning of a term in one taxon group is not always well-defined either. For example, the term "erect" takes on a number of different meanings depending on which botanical thesaurus one consults: the FNA Glossary defines "erect" as a state of orientation, the Oxford Virtual Field Herbarium Plant Characteristics defines it as a state of habit, and two different versions of PATO ontology labeled the concept placement and position respectively [21-23]. Cui conducted a comparison of four machine-readable glossaries in botany (including the above-mentioned three) and found that among 1964 character states extracted from five volumes of FNA and four volumes of FOC, 64 were included by all four glossaries, and only 12 of the 64 were given the same definition by all four glossaries [24]. In the biomedical domain, UMLS (the Unified Medical Language System) is being built since 1986 to bridge different biomedical thesauri. Natural language processing in the biodiversity domain needs a comparable ontological infrastructure. Without consolidating ambiguous definitions, the ability for different annotated collections to communicate with each other is lost, defeating the purpose of semantic annotation.

**Figure 6:**
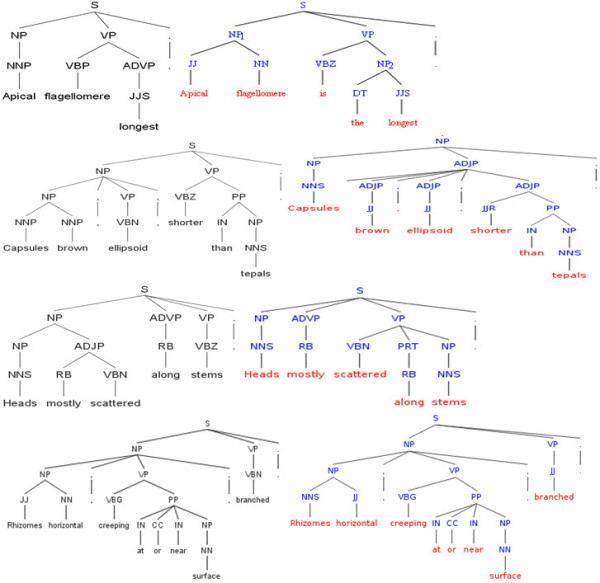
**Parsing trees produced by the Stanford Parser for descriptive sentences**. The first two trees contrast the incorrect parsing of a descriptive sentence in the deviated grammar to the correct parsing of a similar sentence in standard English grammar. The remaining contrasts the incorrect parsing of 3 typical descriptive clauses in the deviated syntax to the correct parsing when the correct Part of Speech (POS) tags were given to the parser. The nodes closest to the words in the parsing trees are the POS tags.

Deviated syntax: Many morphological descriptions are written in a syntax that deviates from standard English syntax (Figure 6 shows some clauses in such a syntax). The syntax makes it difficult to adopt existing natural language parsing tools as part of a semantic annotation system. Syntactic parsers such as the Stanford Parser (SNLP) perform well on sentences using standard grammar, for example, "apical flagellomere is the longest," but not so well for typical sentences in morphological descriptions such as "apical flagellomere longest" (Figure 6). Incorrect parsing at the syntax level will lead to incorrect semantic annotation. There are ways to make modern parsers more useful for biodiversity domain text. Besides retraining a parser with human-annotated domain sentences, one can give the parser useful information directly to guide the parsing. As an exploratory study, a random sample of 20 sentences of different syntactic complexity was parsed using the Stanford Parser (using the Probabilistic Context-Free Grammar: EnglishPCFG) [25]. These sentences include five sentences involving one organ/structure, five sentences involving two organs/structures with at least one preposition, five sentences involving two organs/structures with at least one verb, and five sentences involving three or more organs/structures. Fifteen of the twenty parsing results contain errors. The majority of the errors seem to have stemmed from an incorrect Part of Speech (POS) tag given to a domain term. When corrected POSs were given to the parser, a better parsing resulted for each of the 15 cases, even though a few structural problems remained. Figure 6 shows the improvements that resulted from corrected POS tags for three sentences. The command, the complete set of sentences, and the parsing results can be found in Additional Files 4, 5, and 6. Since general lexicons, e.g., WordNet, for natural language processing, are not very useful (the Stanford Parser uses one of such) and no domain-specific lexicon for biodiversity exists, now the question becomes: where can we find correct POS tags for each of the domain terms?

#### 2. Beneficial characteristics

While the deviated syntax presents a challenge to general-purpose parsers, the fact that the syntax is much simpler than standard English syntax should be considered an advantageous factor. The other two characteristics of morphological descriptions which may be beneficial to a semantic annotation technique are: (1) highly repetitive usage of terms. Morphological descriptions have a very high term repetition rate. Table 2 shows the ratio of the number of unique words to the number of clauses sampled from FNA and Part H of the Treatise of Invertebrate Paleontology [26]. This means that the same term is often used many times in descriptions in different contexts. (2) the availability of parallel text. One taxon is often described and/or redescribed many times. Multiple texts describing the same taxon are called "parallel text" [16]. These two characteristics can be helpful to a semantic annotation system as they provide multiple chances for a system to learn the meaning of a term.

**Table 2:** Word repetition in morphological descriptions.

*source*	*descriptions* *sampled*	*clauses*	*unique* *words*	*unique word* *per clause*
FNA	40	500	614	1.228
FNA	81	1048	834	0.800
FNA	942	12500	1959	0.157
Treatise Part H	2038	9760	2583	0.265

### An unsupervised learning method

With the understanding of challenging and beneficial characteristics of morphological descriptions, Cui explored an unsupervised learning method that discovered organ names and character states directly from descriptions, without being limited by any templates. The algorithm takes advantage of the deviated syntax and works without any lexicons, extraction templates, or training examples [27]. Therefore, the algorithm is expected to work on descriptions of any taxon group written in the deviated syntax. This removes or significantly reduces the manual labor required to craft parsers, templates, or training examples on a collection by collection basis. Different from the supervised learning approach, the unsupervised algorithm identifies organ names and character states mentioned in morphological descriptions by bootstrapping between the subjects (which are typically organ names) and the subsequent words (called "boundary words," over 90% of which are character states) in the clauses [28]. To illustrate the idea, for example, the algorithm is primed with knowledge that "petals" is an organ and can be a subject, then when the algorithm comes across the clause "petals absent," the algorithm would infer that "absent" is a state. Knowing that, the algorithm would further infer that "subtending bracts" in "subtending bracts absent" is an organ. By now, the algorithm has learned two new terms: "absent" is a state and "subtending bracts" is an organ. The algorithm continues searching through the descriptions to apply what it has already learned to discover the unknowns, until there is no new discovery to be made. The algorithm takes the advantage of the deviated yet simple syntax and the repetitive usage of the terms in morphological descriptions. While the assumption that clauses all start with an organ name followed by a state is not always true (since the same organ names or states are often repeatedly used in different combinations in descriptions), the chance for them to be discovered has been shown to be very good.

The identification of organ names is sufficient to perform clause level annotation at an accuracy of 92% to 95%. Compared with the supervised algorithm reported in Cui on the same dataset (i.e., 633 descriptions from FNA) on clause level annotation, the unsupervised algorithm achieved better performance, ran five times faster, and eliminated the need for training examples [11]. Notably, the unsupervised algorithm marked up all clauses left out by the supervised learning algorithm due to the inadequate template problem. Organ names and character states learned by the unsupervised algorithm were significantly cleaner and more useful for marking up new descriptions or constructing domain lexicons [27].

The most recent evaluation on several hundred to several thousand descriptions from volume 19 (Asteraceae) of FNA and Part H (Brachiopods) of the Treatises found that 90% of the organ names learned by the algorithm were correct (precision) and that accounts for 80% to 90% of all organ names mentioned in the descriptions (recall). Over 92% to 98% of learned character states were correct and that accounts for 50% to 75% of all character states mentioned in the descriptions [29]. A plant description correctly annotated by the algorithm is shown in Figure 1.

The unsupervised algorithm has two notable limitations. (1) While the algorithm learned organ names and character states with very good precision, the recall of character states was only in the range of 50% to75%. There is hope to further improve the recall by learning from parallel text. Wood et al. showed that the use of parallel text improved the recall threefold [16]. (2) To fully mark up at the character level, the identified character states must be connected to their characters, and the characters to organs. However, characters are rarely explicitly mentioned in the descriptions. For example, in "stems prostrate to erect," the character to which "prostrate" and "erect" belong is only implied. As discussed earlier, "erect" may be a habit, an orientation, a position, or a placement, depending on which source one consults and when. The confusion on the implied characters is a problem for supervised and unsupervised approaches alike, but in supervised learning, a designation is often arbitrarily made (e.g., making "erect" a habit) and fixed in the extraction templates and training examples, so the issue seems to be resolved, until the annotation needs to be merged with another collection where "erect" is an orientation. Without templates and training examples, the unsupervised algorithm could logically group character states of the same character together by their co-occurrence patterns (e.g., "prostrate" and "erect" often appear together, so they are in the same group), and wait for an authority to determine what they really are. It is much easier for a domain scientist to label the group "dark brown," "chestnut-colored," and "greenish-blue" color than annotating hundreds of training descriptions. The co-occurrence patterns may provide some useful clues for an expert or a group of experts to determine a category for the more troublesome terms such as "erect."

### An overall strategy for semantic annotation of biodiversity documents

**Figure 7:**
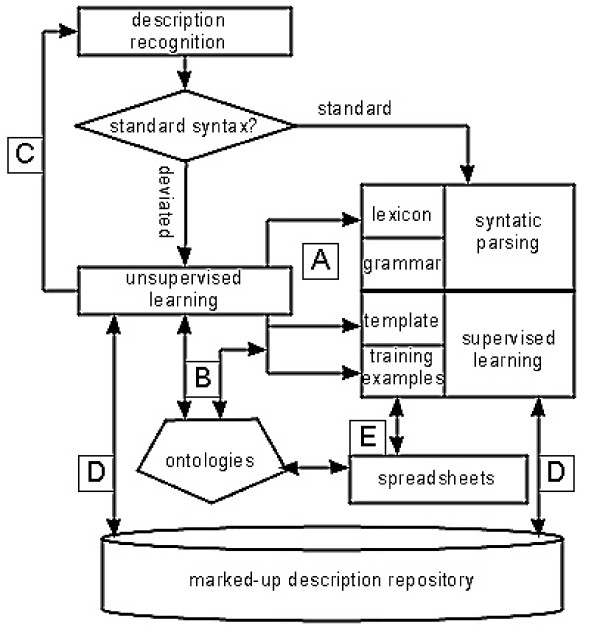
**An overall strategy to automated semantic annotation of morphological descriptions of various taxon groups**.

Having learned characteristics of morphological descriptions and strengths and limitations of existing annotation techniques, in this section we propose an overall strategy for automated semantic annotation of morphological descriptions in general. Figure 7 illustrates the proposed strategy. First, description sections need to be recognized for annotation. If they are in the standard syntax, the existing general-purpose syntactic parsers, in combination with supervised learning methods (not limited to what is reviewed here) are used. If they are in the deviated syntax, the unsupervised learning technique is used.

Since the unsupervised learning technique is cheaper to use than the supervised ones, we propose to process descriptions in the deviated syntax first whenever such an option exists, for the following reasons:

A. Organ names and character states learned by the unsupervised technique can be used to enhance or build domain lexicons. Knowing organ names are nouns and character states are adjectives, most of the parsing errors shown in Figure 6 could be resolved; for example, knowing "flagellomere" is a noun (NN) would correct one of the parsing errors. These concepts can also be used to extend the coverage of the extraction templates used by supervised learning techniques, addressing the problem of inadequate templates. In addition, the cheap yet rather effective unsupervised algorithm may be used to mark up descriptions to obtain "weak" training examples, which can then be refined, if necessary, for supervised learning techniques.

B. The organ names and groups of character states discovered from literature via unsupervised learning may be selected by domain experts to be included in domain ontologies, which in return ensures the annotation produced by any annotation systems is interoperable. Domain knowledge of human experts is best used here, rather than preparing training examples collection by collection.

C. The learned concepts may be used for recognizing and extracting morphological description paragraphs from their parent documents--a necessary first step before morphological information can be annotated further. A description paragraph can simply be distinguished from say, a distribution section, by the density of the words representing organ names and character states. Sophisticated, supervised text classification algorithms have been used for this purpose, but they require training examples to run [30]. We have used the concepts learned unsupervised from a portion of FNA to identify description paragraphs in other volumes with almost effortless 100% accuracy.

In addition:

D. All marked up descriptions should ideally be deposited in a common repository as they can be training examples or otherwise helpful to either supervised or unsupervised learning techniques.

E. Lastly, many systematic biologists are not aware that the spreadsheets they use to draft descriptions could be easily used as training examples for supervised learning. Spreadsheets are another source (besides the literature) of distilled domain knowledge, based on which the meaning of a concept may be verified and determined.

A flexible system architecture such as that provided by GoldenGate, Kepler, or others could be used as the base system where various annotation modules/resources are plug-ins [7,31].

## Discussion

The proposed strategy above is based on the characteristics of several biodiversity document collections we have observed. With millions of pages of biosystematic literature digitized by the Biodiversity Heritage Library and others, systematic biologists, information scientists, and others must work together to put the text into a computer-understandable and interoperable format fast so the knowledge becomes alive again. Language processing infrastructure such as domain lexicons and ontologies should be built and shared not to benefit any particular project but to stay useful for all. As the number of active taxonomists is currently declining, their time should be spent on the most challenging part of the puzzle, namely defining the meaning of domain concepts, so domain ontologies become useful and exert lasting power for a long time to come. A strategy that would lead us to the ultimate goal of a "biodiversity information commons" on the Semantic Web faster involves computer scientists using and developing low-cost unsupervised learning methods for annotating the literature directly or feeding more expensive supervised-learning approaches. But more important than anything else, domain scientists are needed to share their character matrices as training data and to verify learning results produced by the algorithms (including lexicons, ontologies, and annotated documents). Resources should be directed to develop reusable knowledge entities, including benchmarks for evaluating system performances, in standard formats for an accumulative growth of computer-usable knowledge.

## Conclusions

We have experimented with a number of semantic annotation techniques and learned the characteristics of morphological descriptions over time. These experiences have led us to the overall strategy proposed above. With the support of an NSF grant and a group of enthusiastic domain scientists, we are implementing the strategy, including developing the unsupervised learning algorithm and using it to help lexicon and ontology constructions. All will be further developed and tested on different taxon groups for character-level annotation and released for public download by 2011. Post-2011 we plan to make use of the lexicons and ontologies produced to annotate biodiversity-related, true natural language text. Along the way we hope to develop standard benchmark datasets for algorithm evaluation in the biodiversity domain.

## Supplementary Material

Additional file 1 **domainConceptsExtractedFromPartVTreatise**. Contains concepts automatically extracted from Part V of the Treatise on Invertebrate Paleontology. Click here for file

Additional file 2 **domainConceptsExtractedFromFNA**. Contains concepts automatically extracted from 2300+ descriptions of FNA. Click here for file

Additional file 3 **domainConceptsExtractedFromFOC**. Contains concepts automatically extracted from 13,000+ descriptions of FOC Click here for file

Additional file 4 **lexparserPOS**. Executable Windows commandline commands for running the Stanford Parser. To run the command after installing the Parser, type the following on the commandline: lexparserPOS.bat "testsentPOS.txt". testsentPOS.txt is the Additional file 5. Click here for file

Additional file 5 **testsentPOS**. Contains the 20 test sentences for the Stanford Parser. Sentences are listed in the order of complexity. Sentences parsed incorrectly by the Stanford Parser are listed at least twice, one of which showing the POS tags assigned by the Stanford Parser, and the others showing correct alternative POS tags. POS-tagged by the Stanford Parser and with corrected POS tags. Use this file to run the command listed in Additional file 4 to obtain the contrasting parsing results for each sentence. The results are not shown as "trees." To generate parsing trees, use the service provided at http://ironcreek.net/phpsyntaxtree/ (need to all ( ) to []) Click here for file

Additional file 6 **resultPOS**. Contains the contrasting parsing results for the 15 sentences parsed incorrectly by the Stanford Parser. Click here for file
